# Clinical Course of COVID-19 Disease in Children Treated With Neoplastic Diseases in Hungary

**DOI:** 10.3389/pore.2022.1610261

**Published:** 2022-03-31

**Authors:** Judit Müller, Dóra Szűcs-Farkas, István Szegedi, Monika Csóka, Miklós Garami, Lilla Györgyi Tiszlavicz, Péter Hauser, Gergely Kriván, Krisztina Csanádi, Gábor Ottóffy, Béla Nagy, Csongor Kiss, Gábor Kovács

**Affiliations:** ^1^ Second Department of Pediatrics, Semmelweis University, Budapest, Hungary; ^2^ Department of Pediatrics, Faculty of Medicine, University of Debrecen, Debrecen, Hungary; ^3^ Department of Pediatrics, University of Szeged, Szeged, Hungary; ^4^ Haematology/Oncology and Pediatric Bone Marrow Transplantation Unit, Child Health Centre, Borsod-Abauj-Zemplen County Hospital, Miskolc, Hungary; ^5^ Pediatric Bone Marrow Transplantation Department, South-Pest Centrum Hospital, Budapest, Hungary; ^6^ Hemato-Oncology Unit, Heim Pal Children’s Hospital, Budapest, Hungary; ^7^ Department of Pediatrics, University of Pecs, Pecs, Hungary; ^8^ Department of Laboratory Medicine, Faculty of Medicine, University of Debrecen, Debrecen, Hungary

**Keywords:** SARS-CoV-2, COVID-19, pediatric malignancy, chemotherapy delay, SARS-CoV-2S antibodies

## Abstract

We report on children with cancer in Hungary suffering from COVID-19, surveying a 13-months-long period of time. We performed a retrospective clinical trial studying the medical documentation of children treated in seven centers of the Hungarian Pediatric Oncology-Hematology Group. About 10% of children admitted to tertiary hemato-oncological centers for anti-neoplastic treatment or diagnosis for *de novo* malignancies were positive for SARS-CoV-2 infection. Nearly two-thirds of the infected patients were asymptomatic or had only mild symptoms but showed seropositivity by 1–4.5 months after positive PCR. One third of the SARS-CoV-2-positive children were hospitalized due to symptomatic COVID-19. Five children required antiviral treatment with remdesivir. One child was referred to the intensive care unit, requiring intubation and mechanical ventilation. Delay in the scheduled anti-cancer treatment did not exceed 2 weeks in the majority (89%) of cases. There was only one patient requiring treatment deferral longer than a month. There was no COVID-19-related death in patients under 18 years of age, and nor was multisystem inflammatory syndrome diagnosed. In conclusion, SARS-CoV-2 infection did not represent an untoward risk factor among children with cancer in Hungary.

## Introduction

The current pandemic of the coronavirus disease 2019 (COVID-19) affects children and adults worldwide [1]. Severe forms and late consequences seem to be less frequent among children than in adults [2–4]. However, few data have been published on severity and disease outcome of children with hematologic malignancies and solid tumors [5–7]. Cancer and COVID-19 may each deteriorate the course of the other. Immunity of children with cancer is compromised which may increase the risk of acquiring infections and many infectious diseases were shown to exhibit a less favorable outcome than in otherwise healthy persons [5,6]. At the onset of the COVID-19 pandemic it was not known if SARS-CoV-2 infection *per se* or due to its complications may have an adverse effect on the clinical course of the underlying malignant disease. Another potential hazard of COVID-19, in particular of moderately severe and severe cases, is that it may result in a delay of the scheduled anti-cancer treatment, thereby decreasing the chance for a cure. One year ago, pediatric oncologist-hematologists did not have sufficient experience and guidance regarding treating children with concomitant cancer and COVID-19. Initial data from China suggested that concomitant COVID-19 adversely affected adult patients with cancer [7]. The first report on COVID-19 in a pediatric bone-marrow transplant and hematology-oncology center was published from Italy a few months later.

Here we report on observations on SARS-CoV-2 infection and COVID-19 among children with cancer diagnosed and treated in tertiary pediatric cancer centers of the Hungarian Pediatric Oncology-Hematology Group (HPOG). We investigated the incidence of SARS-CoV-2 infection in children with newly diagnosed cancer and those currently undergoing anti-cancer therapy. Clinical course of COVID-19 and potential effects on the applied anti-cancer treatment were registered and evaluated.

## Methods

Data obtained from each consecutive patient admitted to any of the seven tertiary pediatric cancer treatment centers of HPOG were investigated in this retrospective study. Data relevant to this research were collected from electronic patient data files. We compared the cumulative number of the hospitalized pediatric cases (age <19 years) between March 2020 and April 2021 with the same period of the previous year (i.e., March 2019 to March 2020). As per legal regulation, all children admitted with a suspicion of a neoplastic disorder and children with already diagnosed cancer were tested by oropharyngeal swab samples with COVID-19 specific reverse transcription polymerase chain reaction (RT-qPCR) analysis regardless of the presence or absence of COVID-19-specific symptoms [8]. In 10 randomly selected subjects, automated Cobas ®SARS-CoV-2 serology tests (Roche Diagnostics, Mannheim, Germany) measuring total immunoglobulin (Ig) levels against nucleocapsid (N) and spike-protein1 receptor binding domain (S1-RBD)- specific antibodies in serum samples were also performed. Due to the former protocol of the routine laboratory, anti-SARS-CoV-2 S1-RBD titers were determined up to 250 U/ml and sera were not diluted to determine higher absolute levels. Seropositivity was considered according to the official cut-off value of these tests [anti-N: ≥ 1.0 (COI, cut-off index); anti-S1-RBD: ≥0.8 U/mL]. In the cases of three patients, anti-N total Ig levels were not available. Importantly, none of these patients received any vaccination against SARS-CoV-2, thus a positive serology result was produced by the SARS CoV-2 infection itself.

Age and gender of patients, the severity of COVID-19, and any delays in scheduled treatments were registered. We examined the correlations between the symptoms of the SARS-CoV-2 PCR-positive patients and the treatment of the underlying disease and we sub-grouped them by gender and age. This study was approved by the Hungarian Scientific and Research Ethics Committee (TUKEB, No IV/5736-3/2021/EKU).

## Results

**FIGURE 1:**
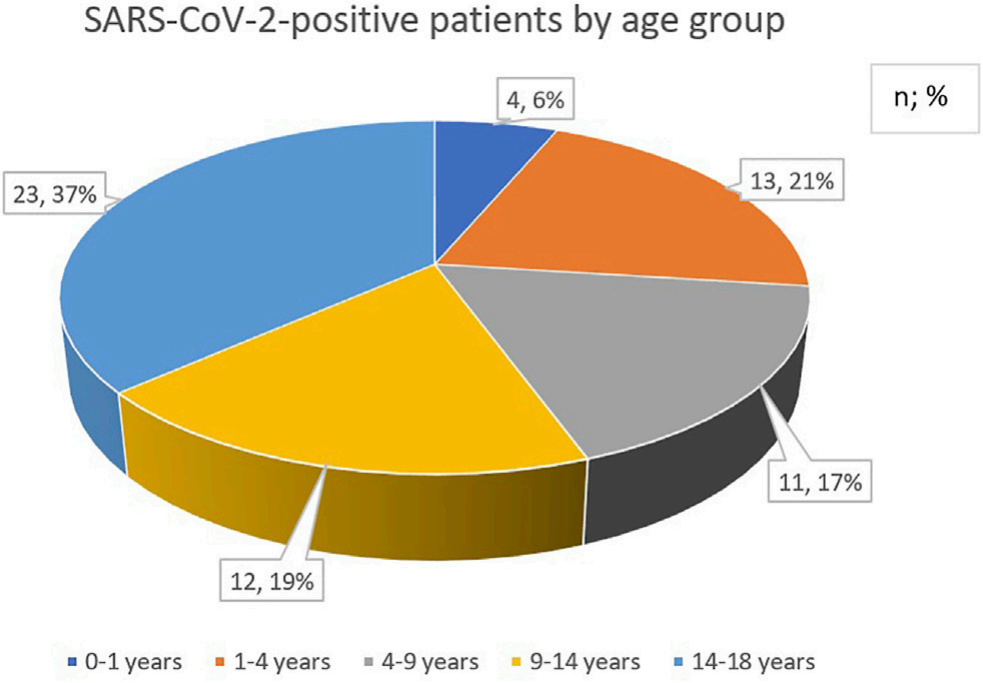
Age distribution of pediatric cancer patients with SARS-CoV-2 infection.

During the time of monitoring, a total of 603 children were admitted of whom 341 (57%) had newly diagnosed cancer. Sixty-three of 603 patients (10%) proved to be positive for SARS-CoV-2 infection on admission. From these 63 patients, 30 (48%) were females and 33 (52%) were males. The age distribution of SARS-CoV-2-positive children with cancer showed a slight shift towards older age groups of patients being 9-years-of-age and older (Figure 1).

Nearly two-thirds of the infected patients, 39 (62%) were asymptomatic or had mild symptoms only. Among them, 3 (8%) were under 1 year of age, 8 (20%) were between 1 and 4 years of age, 5 (13%) were between 4 and 9 years of age, 14 (36%) children were between 9 and 14 years of age and 9 (23%) patients were over 14 years of age. Fourteen of the 63 patients (22%) recovered from COVID-19 within 1–2 weeks. One of these children was under 1 year of age, 3 individuals were between 1 and 4 years of age, 3 children were between 4 and 9 years, 2 patients were between 9 and 14 years, and 5 (36%) were over 14 years of age. Eight patients (13%) required more than 2 weeks for total convalescence from COVID-19. From these patients two were between 1 and 4 years of age, one patient was between 9 and 14 years of age, and 5 patients were over 14 years of age. Five children (8%) required antiviral treatment with remdesivir. One of these 5 patients was between 1 and 4 years of age and the other 4 children were over 14 years of age.

Twenty children (32%) were hospitalized due to COVID-19. Four children were between 1 and 4 years of age, 7 were between age of 4 and 9 years of age, 3 were between 9 and 14 years of age and 6 patients were over 14 years of age. From these 20 patients, one was referred to the intensive care unit requiring intubation and mechanical ventilation. This child was 5 years of age as shown in Table 1. There was no lethal case. Multisystem inflammatory syndrome was not registered in this study.

**TABLE 1:** Clinical data of SARS-CoV-2 positive children by age groups (ICU: intensive care unit).

	No of Children
Asymptomatic or mild symptoms	39
0–1 year	3
1–4 years	8
4–9 years	5
9–14 years	14
14–18 years	9
Recovery within 1–2 weeks	14
0–1 year	1
1–4 years	3
4–9 years	3
9–14 years	2
14–18 years	5
Recovery more than 2 weeks	8
0–1 year	0
1–4 years	2
4–9 years	0
9–14 years	1
14–18 years	5
Antiviral (remdesivir) therapy	5
0–1 year	0
1–4 years	1
4–9 years	0
9–14 years	0
14–18 years	4
Hospitalization needed	20
0–1 year	0
1–4 years	4
4–9 years	7
9–14 years	3
14–18 years	6
ICU needed	1
0–1 year	0
1–4 years	0
4–9 years	1
9–14 years	0
14–18 years	0

Of the 63 patients with PCR-proven SARS-CoV-2 infection there were 29 cases which did not require a delay or less than 1-week postponement in the scheduled anti-cancer treatment. Four of these patients were under 1 year of age, 7 were between 1 and 4 years of age, 4 were between 4 and 9 years of age, 4 subjects were between 9 and 14 years of age, and 10 patients were over 14 years of age. Twenty-eight patients (44%) had 1–2 weeks long treatment delay. Of these 28 patients six children were between 1 and 4 years of age, 7 were between 4 and 9 years of age, 6 were between 9 and 14 years of age, and 9 patients were over 14 years of age. In 6 cases there were a 3–4 weeks long treatment delay. All of these patients were older than 14 years of age. There was only one 14-year-old patient requiring treatment deferral of more than a month due to COVID-19. Thirty-seven (59%) of SARS-CoV-2-positive patients received intensive intravenous or intrathecal chemotherapy, 14 (22%) of patients were on oral maintenance therapy and 11 (17%) children started their treatment with newly diagnosed cancer at the time of diagnosing the SARS-CoV-2 infection. One patient received immunotherapy.

In case of 10 randomly selected patients, SARS-CoV-2 serology tests were also performed to analyze if antibodies could be detected against N-and S1-RBD-protein in response to SARS-CoV-2 infection in the presence of a malignant co-morbidity. Blood sampling for serology occurred between 1 and 4.5 months after the onset of COVID-19. Among these patients, six had acute lymphoblastic leukemia, and the other subjects were previously diagnosed with non-Hodgkin lymphoma, anaplastic medulloblastoma, and rhabdomyosarcoma (Table 2). Most of these patients were asymptomatic or exhibited a mild course of COVID-19. Six patients were seropositive demonstrating both elevated anti-N and anti-S1-RBD total Ig concentrations, while four children showed undetectable Ig levels (Table2). Regarding the interval between PCR and serology, these later results were not caused by different timings compared to that of others.

**TABLE 2:** Demographic and clinical data of SARS-CoV-2-positive children with serology test results (ALL, acute lymphoblastic leukemia; MBL, anaplastic medulloblastoma; NHL, non-Hodgkin lymphoma; RMS, rhabdomyosarcoma; COI, cut-off index; n/a, not available; n.d., not determined).

Patient No	Age (years)	Gender (F/M)	Time Between PCR and Serology (months)	Symptoms of COVID-19	Anti SARS-CoV-2 N total Ig (COI)	Anti-SARS-CoV-2 S1-RBD total Ig (U/mL)	Malignancy
1	7	F	n/a	no symptoms	117.5	184.6	ALL
2	11	M	1.5	mild symptoms	0.084	<0.4	NHL
3	6	M	2	mild symptoms	8.74	146.0	MBL
4	2	M	4.5	no symptoms	0.074	<0.4	RMS
5	1	F	4	mild symptoms	153.1	>250.0	ALL
6	6	F	2	no symptoms	140.0	>250.0	ALL
7	15	M	1.75	no symptoms	0.401	16.1	NHL
8	17.5	M	1	no symptoms	n.d.	222.8	ALL
9	16	F	4	no symptoms	n.d.	<0.4	ALL
10	16	M	5	no symptoms	n.d.	<0.4	ALL

## Discussion

In a country with a population of nearly 10 million people (population size of Hungary in 2020: 9.76 million, with 1.7 million children <18 years of age), 63 children with cancer have been diagnosed with SARS-CoV-2 infection by nasal swab RT-qPCR in a period of 13 months [9]. This figure is about 10% of all children with cancer (603 patients including 341 *de novo* cases) managed in seven tertiary pediatric oncology-hematology treatment centers of the Hungarian Pediatric Oncology-Hematology Group [10]. The majority of patients (56%) were 9-years-of-age or older which is in contrast to the age distribution of pediatric cancer in Hungary and worldwide [10]. Here we reported, for the first time in Central Europe, on a comprehensive evaluation of the clinical course of SARS-CoV-2 infection among children with cancer, who were tested for the infection. A further strength of this study is the participation of all Hungarian tertiary pediatric oncology-hematology centers in this investigation that provides a nationwide coverage. The relatively small size of the investigated population may represent a fundamental flow of the study which may bias the interpretation of the results. However, the number of patients is proportional to the size of the population of Hungary. The retrospective nature is a further limitation of this study.

The first two cases of COVID-19 were confirmed in Hungary on March 4, 2020 among graduate university students returning from their home countries. One week later an emergency regime was introduced by the government and soon, restrictions were tightened further. Extensive testing procedures were introduced to reduce the risk of SARS-CoV-2 infection in the medical departments, especially in pediatric oncology-hematology wards with a special focus not only on patients but also on accompanying caregivers and health care personnel. Strict protective measures were encouraged, such as emphasizing frequent and thorough hand washing, using proper personal protective equipment, social distancing, and regular disinfection of surfaces. Only one caregiver per patient was allowed to accompany children admitted to inpatient units. Unscreened patients and caregivers were separated in an isolated part of the hospital. RT-qPCR swab tests to detect SARS-CoV-2 were performed in each case of children with cancer and their accompanying caregivers regardless of symptoms, COVID-19 contact status, and type of planned interventions.

Due to the protective measures employed nationwide as well as in the hospitals, including pediatric oncology-hematology units, the COVID-19 pandemic did not exert a major negative impact on children with cancer in Hungary. The number of newly diagnosed children with cancer did not differ substantially between the investigated period and the preceding 1 year before the COVID-19 pandemic. However, there was a shift from inpatient to outpatient care noticing a 20% reduction in the number of hospitalized patients. The majority of Hungarian pediatric cancer patients with SARS-CoV-2 infection was asymptomatic or exhibited mild symptoms with a skew towards the younger age groups. One third (20 patients) of the Hungarian cohort were admitted to hospital units dedicated to COVID-19 treatment because of symptomatic COVID-19. Implementation of antiviral therapy was necessary in five of the patients hospitalized because of COVID-19, and one patient was managed in an intensive care unit requiring ventilatory support. No deaths attributed to COVID-19 were reported. The number of patients requiring either anti-viral treatment or ventilatory support was fortunately small. We think that the interpretation of a proper antiviral treatment approach of children with cancer and coinciding COVID-19 would require a much larger sized clinical study. Moreover, SARS-CoV-2 infection did not substantially influence the delivery of scheduled anticancer treatment. Treatment delay did not exceed 2 weeks in the majority (89%) of cases and no therapy was omitted.

Few observations have been published on concomitant SARS-CoV-2 infection and cancer among pediatric patients so far. The majority of publications on the characteristics and sequela of SARS-CoV-2 infection in children with cancer reported on similar data as the present Hungarian investigation. SARS-CoV-2 infection in the majority of this patient group was asymptomatic or was accompanied by mild symptoms as reported from France, Lombardy, Italy, Moscow, Russia, New York City, New York-New Jersey, U.S., and the United Kingdom [11–17]. These individual centers and centers working in the above regions and countries, similar to our results, considered that SARS-CoV-2 infection did not severely affect children with cancer, nor did it interfere considerably with their treatments. In contrast to these findings, a Turkish investigation reported on a dramatic decrease in the number of both *de novo* diagnosed and treated pediatric cancer patients [18]. Participants of the St Jude Global Alliance and International Society for Paediatric Oncology survey also reported on a reduction of newly diagnosed patients and gross interruptions, even complete abandonment of anticancer therapies delivered [19–21]. According to this group of investigators, severe and critical conditions of COVID-19 were observed in one fifth of patients and deaths occurred in a higher proportion than were reported in the literature surveying the general pediatric population. However, these disturbing consequences affected mainly low- and middle-income countries [22].

An important added value of the Hungarian study was the serologic investigation of a subgroup of pediatric cancer patients confirmed with SARS-CoV-2 infection. Although the number of the study patients is small, we were able to demonstrate a seroconversion with the development of increased anti-SARS-CoV-2 N and S1-RBD antibodies in the majority (6/10) of patients. However, four patients did not have measurable levels of antibodies. Similar investigation was published only by the Moscow group. They investigated a larger number of patients in multiple timepoints and found a 100% seroconversion after 6-weeks which started to decrease after three consecutive weeks to 54% by week 18, similar to our findings [14]. These results indicate that pediatric cancer patients, immunocompromised due to their underlying malignancy and its treatment, are able to mount a humoral immune response against the SARS-CoV-2 infection, however, this natural immunity may decline four to 5 months after harboring the infection leaving patients unprotected against a reinfection in a new wave of epidemics without vaccination. We have to note that additional patients need to be involved for the follow-up of anti-SARS-CoV-2 antibodies to investigate the dynamic of humoral response in children with cancer.

### Conclusion

During the study period overlapping with the COVID-19 epidemics in Hungary, a Central-European country, SARS-CoV-2 positivity was detected in 10 percent of children with cancer. The course of SARS-CoV-2 infection did not result in more severe cases among these patients than in the general population. Although there was a shift from inpatient to outpatient management and minor treatment delays not exceeding 2 weeks were noticed, COVID-19 epidemics did not present an unacceptable hurdle for pediatric oncology-hematology care in Hungary.

## Data Availability

The raw data supporting the conclusion of this article will be made available by the authors, without undue reservation.

## References

[1] Pollard CA Morran MP Nestor-Kalinoski AL. The COVID-19 Pandemic: a Global Health Crisis. Physiol Genomics (2020) 52(11):549–57. 10.1152/physiolgenomics.00089.2020 32991251 PMC7686876

[2] Dong Y Mo X Hu Y Qi X Jiang F Jiang Z Epidemiology of COVID-19 Among Children in China. Pediatrics (2020) 145:e20200702. 10.1542/peds.2020-0702 32179660

[3] Tezer H Bedi̇r Demi̇rdağ T. Novel Coronavirus Disease (COVID-19) in Children. Turk J Med Sci (2020) 50(SI-1):592–603. 10.3906/sag-2004-174 32304191 PMC7195991

[4] Guo C-X He L Yin J-Y Meng X-G Tan W Yang G-P Epidemiological and Clinical Features of Pediatric COVID-19. BMC Med (2020) 18(1):250. 10.1186/s12916-020-01719-2 32762696 PMC7408975

[5] Loeffen EAH Knops RRG Boerhof J Feijen EAM Merks JHM Reedijk AMJ Treatment-related Mortality in Children with Cancer: Prevalence and Risk Factors. Eur J Cancer (2019) 121:113–22. 10.1016/j.ejca.2019.08.008 31569066

[6] Inaba H Pei D Wolf J Howard SC Hayden RT Go M Infection-related Complications during Treatment for Childhood Acute Lymphoblastic Leukemia. Ann Oncol (2017) 28(2):386–92. 10.1093/annonc/mdw557 28426102 PMC5834143

[7] Liang W Guan W Chen R Wang W Li J Xu K Cancer Patients in SARS-CoV-2 Infection: a Nationwide Analysis in China. Lancet Oncol (2020) 21:335–7. 10.1016/S1470-2045(20)30096-6 32066541 PMC7159000

[8] National Public Health Center. National Public Health Center (2020). Available from: https://www.nnk.gov.hu/attachments/article/567/Aktualiz%C3%A1lt%20elj%C3%A1r%C3%A1srend%2011.07.pdf .

[9] Hungarian Central Statistical Office. Population of Hungary by Sex and Age, 1 January. Hungarian Central Statistical Office (2021). Available from: https://www.ksh.hu/stadat_files/nep/hu/nep0003.html (Accessed 06 21, 2021).

[10] Schuler D. Systemizing Childhood Cancer Care in Hungary: Twenty-Five Years of Progress. Med Pediatr Oncol (1999) 32:68–70. 10.1002/(sici)1096-911x(199901)32:1<68::aid-mpo16>3.0.co;2-3 9917759

[11] André N Rouger‐Gaudichon J Brethon B Phulpin A Thébault É Pertuisel S COVID‐19 in Pediatric Oncology from French Pediatric Oncology and Hematology Centers: High Risk of Severe Forms? Pediatr Blood Cancer (2020) 67:e28392. 10.1002/pbc.28392 32383827 PMC7235489

[12] Balduzzi A Brivio E Rovelli A Rizzari C Gasperini S Melzi ML Lessons after the Early Management of the COVID-19 Outbreak in a Pediatric Transplant and Hemato-Oncology center Embedded within a COVID-19 Dedicated Hospital in Lombardia, Italy. Estote Parati. Bone Marrow Transpl (2020) 55(10):1900–5. 10.1038/s41409-020-0895-4 PMC7167532 32313181

[13] Bouffet E Challinor J Sullivan M Biondi A Rodriguez‐Galindo C Pritchard‐Jones K. Early Advice on Managing Children with Cancer during the COVID‐19 Pandemic and a Call for Sharing Experiences. Pediatr Blood Cancer (2020) 67:e28327. 10.1002/pbc.28327 32239747

[14] Mayanskiy N Luchkina P Fedorova N Lebedin Y Ponomareva N. Seroconversion and Dynamics of the Anti-SARS-CoV-2 Antibody Response Related to a Hospital COVID-19 Outbreak Among Pediatric Oncology Patients. Leukemia (2021) 35(6):1820–2. 10.1038/s41375-021-01288-0 34007047 PMC8129958

[15] Boulad F Kamboj M Bouvier N Mauguen A Kung AL. COVID-19 in Children with Cancer in New York City. JAMA Oncol (2020) 6(9):1459–60. 10.1001/jamaoncol.2020.2028 32401276 PMC7221844

[16] Madhusoodhan PP Pierro J Musante J Kothari P Gampel B Appel B Characterization of COVID-19 Disease in Pediatric Oncology Patients: The New York-New Jersey Regional Experience. Pediatr Blood Cancer (2021) 68(3):e28843. 10.1002/pbc.28843 33338306 PMC7883045

[17] Millen GC Arnold R Cazier J-B Curley H Feltbower RG Gamble A Severity of COVID-19 in Children with Cancer: Report from the United Kingdom Paediatric Coronavirus Cancer Monitoring Projec. Br J Cancer (2021) 124:754–9. 10.1038/s41416-020-01181-0 33299130 PMC7884399

[18] Kutluk MT Ahmed F Kirazli M Bajin IY Müngen E Ekinci S The Effect of the COVID-19 Pandemic on Paediatric Cancer Care: Lessons Learnt from a Major Paediatric Oncology Department in Turkey. Ecancermedicalscience (2021) 15:1172. 10.3332/ecancer.2021.1172 33680086 PMC7929778

[19] Graetz D Agulnik A Ranadive R Vedaraju Y Chen Y Chantada G Global Effect of the COVID-19 Pandemic on Paediatric Cancer Care: a Cross-Sectional Study. Lancet Child Adolesc Health (2021) 5:332–40. 10.1016/S2352-4642(21)00031-6 33675698 PMC7929816

[20] Mukkada S Bhakta N Chantada GL Chen Y Vedarajus Y Faughnan L Global Characteristics and Outcomes of SARS-CoV-2 Infection in Children and Adolescentswithcancer (GRCCC): a Cohortstudy. Lancet Oncol (2021) 22(10):1416–26. 10.1016/S1470-2045(21)00454-X 34454651 PMC8389979

[21] Parambil BC Moulik NR Dhamne C Dhariwal N Narula G Vora T COVID-19 in Children with Cancer and Continuation of Cancer-Directed Therapy during the Infection. Indian J Pediatr (2021) 2021:1. 10.1007/s12098-021-03894-3 PMC8354680 34378149

[22] Liu C Zhao Y Okwan-Duodu D Basho R Cui X. COVID-19 in Cancer Patients: Risk, Clinicalf Eatures, and Management. Cancer Biol Med (2020) 17(3):519–27. 10.20892/j.issn.2095-3941.2020.0289 32944387 PMC7476081

